# Acaricidal Effect of Essential Oils in the Control of *Rhipicephalus microplus* and *Amblyomma mixtum* Larvae in Mexico

**DOI:** 10.3390/pathogens15040403

**Published:** 2026-04-08

**Authors:** Juan Manuel Hernández-Domínguez, Roberto González-Garduño, Edgar Castro-Saines, Rodolfo Lagunes-Quintanilla, Roger Iván Rodríguez-Vivas, Agustín Olmedo-Juárez, Jorge Alberto Cortes-Morales, Claudia Yesenia León-González

**Affiliations:** 1 Unidad Regional Universitaria SurSureste, Universidad Autónoma Chapingo, Teapa 86800, Tabasco, Mexico; al26510202@chapingo.mx (J.M.H.-D.); al26510235@chapingo.mx (C.Y.L.-G.); 2 Centro Nacional de Investigación Disciplinaria en Salud Animal e Inocuidad, Instituto Nacional de Investigaciones Forestales, Agrícolas y Pecuarias, Jiutepec 62550, Morelos, Mexico; castro.edgar@inifap.gob.mx (E.C.-S.); rodolfo.lagunes@gmail.com (R.L.-Q.); aolmedoj@gmail.com (A.O.-J.); 3 Facultad de Medicina Veterinaria y Zootecnia, Universidad Autónoma de Yucatán, Mérida 97700, Yucatán, Mexico; rvivas@correo.uady.mx; 4 Escuela de Estudios Superiores del Jicarero, Universidad Autónoma del Estado de Morelos, Carretera Galeana-Tequesquitengo s/n, Colonia El Jicarero, Jojutla 62909, Morelos, Mexico; ing_cortesmorales@yahoo.com.mx

**Keywords:** *Amblyomma mixtum*, essential oils, *Rhipicephalus microplus*, ticks

## Abstract

The objective of this study was to evaluate essential oils (EOs) as an alternative control method for *Rhipicephalus microplus* and *Amblyomma mixtum* larvae. The EOs used were obtained by steam distillation from the leaves of cinnamon (*Cinnamomum verum*), mexican mint (*Plectranthus amboinicus*), lemongrass (*Cymbopogon citratus*), peppercorns (*Pimenta dioica*), and peruvian pepper tree (*Schinus molle*). To evaluate the acaricidal effect of EOs against the larvae of two tick species, a larval immersion test (LIT) was performed using six concentrations (10 mg/mL, 5 mg/mL, 2.5 mg/mL, 1.87 mg/mL, 1.25 mg/mL, and 0.6 mg/mL), in addition to a negative control group (water + ethanol) and three positive controls (organophosphate, formamidine, and pyrethroid). LIT results were obtained 48 h after exposure. Data were processed using Probit procedure to determine the lethal concentrations at 50% (LC_50_), 95% (LC_95_), and 99% (LC_99_). For *R. microplus*, 99% mortality was obtained at concentrations as low as 1.4 mg/mL for *S. molle*, while the highest LC_99_ was recorded with *P. dioica* at 23 mg/mL. In the case of *A. mixtum*, higher concentrations were required to achieve a high mortality rate. EO of *P. amboinicus* had the lowest acaricidal effect, requiring 26.2 mg/mL to achieve an LC_99_, while *S. molle* required a concentration of 6.9 mg/mL to achieve an LC_99_.

## 1. Introduction

Livestock farming is an economically important activity in Mexico [1,2], generating 3.5% of the national gross domestic product (GDP) and 56% of the agricultural GDP [3]. Furthermore, it provides high-quality protein in products such as meat, milk, and dairy products. One of the main challenges facing livestock farming in the tropics is health management and specifically tick infestations [4,5] because they cause a significant economic impact [6] due to the direct effects of feeding on the host’s blood and causing weight loss and damage to the hide, and also due to indirect effects such as the transmission of bloodborne pathogens [7], among which the hemoparasite *Babesia* sp. and the rickettsia *Anaplasma* sp. stand out [8].

External parasites cause losses estimated in billions of dollars in tropical and subtropical regions worldwide [9], with ticks being the most significant ectoparasites affecting livestock production. Although ticks can also parasitize amphibians, reptiles, birds, and mammals, they are hematophagous in all their life stages [10]. Globally, approximately 900 tick species have been reported, belonging to three families: Ixodidae, Argasidae, and Nuttalliellidae. In Mexico, only 100 species have been described, belonging to the Ixodidae and Argasidae families [11], which are the two most important tick families in the Americas [12]. Some species require one to three vertebrate hosts to complete their life cycle, while others can parasitize several hosts. The effects on animal health are numerous, causing anorexia, toxicosis, blood loss, stress and general irritation, decreased productivity, suppressed immune function, and skin damage [13]. Furthermore, they can inoculate parasites such as *Babesia bovis*, *B. bigemina*, and rickettsiae like *Anaplasma marginale* or *Anaplasma centrale* through their saliva. Added to this are economic losses due to treatment costs [14], resulting in estimated economic losses of between US$13.9 and US$18.7 billion annually worldwide [15]. Due to their potential to transmit secondary diseases, six genera of ticks from the Ixodidae family (*Amblyomma* spp., *Dermacentor* spp., *Haemaphysalis* spp., *Hyalomma* spp., *Ixodes* spp., and *Rhipicephalus* spp.) and two from the Argasidae family (*Ornithodorus* spp. and *Otobius* spp.) are of veterinary importance worldwide [16].

In Mexico, cattle infestation with *R. microplus*, the main tick species affecting cattle and distributed across 65.9% of Mexican territory [17], causes annual economic losses of around US$578 million [18]. *A. mixtum* is the second-most important tick in Mexican livestock and is found in 609,857 km^2^ (31% of the total area) of tropical, arid, and temperate zones [19].

Tick control has primarily relied on the frequent use of commercial chemical acaricides, which can cause resistance in the parasite, as well as toxicity and environmental hazards [20]. The development of acaricide resistance has been reported in many chemicals used for tick control in tropical and subtropical countries [21]. Given the problems caused by the continuous use of conventional products, there has been a growing interest in alternative control methods, including the evaluation of secondary metabolites from various plants on economically important arthropods in the agricultural sector [22]. Plants contain compounds derived from secondary metabolism that belong to one of five main groups: phenolics, alkaloids, saponins, terpenes, and glycosides [23]. Secondary compounds are attributed with antiparasitic properties, and many of these are obtained as extracts and essential oils (EOs). For this reason, the use of metabolites has represented an alternative for tick control, since several plant compounds repel these parasites, decrease their reproductive potential, and reduce their survival rate [24]. EOs have shown significant activity against all life stages of economically important tick species [25]. Essential oils from multiple plant species have been evaluated for their acaricidal potential [26] against various tick life stages, studying their mechanical and repellent effects, as well as their mechanism of action. Although there are a wide variety of plants that produce essential oils, this study focused on those that are abundant in the region to reduce the cost of production. In addition, plants with at least one document on their effect against ticks were sought; therefore, the following oils were selected: cinnamon (*Cinnamomum verum)*, peppercorns (*Pimenta dioica*), peruvian pepper tree (*Schinus molle*), lemon grass (*Cymbopogon citratus*) and mexican mint (*Plectranthus amboinicus*) in the sustainable control of ticks [5,27,28,29,30].

In addition to confirming the action of the selected EOs on the tick *Rhipicephalus microplus*, the study sought to explore their action on *Amblyomma mixtum*, another highly prevalent species in the region, of significant economic importance due to the multiple damages it causes [11] and with few reference studies in warm climates.

Therefore, the hypothesis was that essential oils possess metabolites with an acaricidal effect that causes mortality of larvae of the two main tick species in the tropics of Mexico, and the objective of this study was to verify the mortality of some essential oils on larvae of the tick *Rhipicephalus microplus* and to evaluate the same concentrations on *Amblyomma mixtum* to determine the acaricidal effect of the EOs as a sustainable control alternative.

## 2. Materials and Methods

### 2.1. Location

The processing of plant material and bioassays of *A. mixtum* were performed at the Animal Health Laboratory of the South-Southeast Regional University Unit of the Autonomous University of Chapingo (URUSSE-UACh), located in San José Puyacatengo, in the municipality of Teapa, Tabasco, Mexico. The bioassays of the *R. microplus* strain was carried out at the Arthropodology Laboratory of the National Center for Disciplinary Research in Animal Health and Safety (CENID-SAI) of the National Institute of Forestry, Agricultural and Livestock Research (INIFAP), located in Jiutepec, Morelos, Mexico.

### 2.2. Biological Material

Different anatomical structures (fruits, leaves or stems) from five plants with a history of using their essential oils in animal or human health were used. Plant material was collected in the municipalities of Salto de Agua (Chiapas state); Teapa (Tabasco, state), and Temascalapa (State of Mexico). For EO extraction from foliage, the leaves were separated from the woody material. For extraction from seeds, such as those of Peruvian pepper tree (*Schinus molle*) and allspice (*Pimenta dioica*), the samples were cleaned by removing pieces of branches and leaves (Table 1). The collected plant species were compared against those in the repository of the National Herbarium of Mexico, resulting in the following catalog numbers [31].

The plants were collected over a period of one month (February to March 2024) and only one extraction batch was taken from each plant. After cleaning, the material was placed in a drying oven at 60 °C for 48 h for foliage and 76 h for materials with higher moisture content (fruits). The initial weight of the sample was recorded, and after reaching a constant weight, the final weight of each paper bag was recorded to determine the dry matter content. The dried plant material was then ground to obtain particles smaller than 1 mm and stored in plastic containers labeled with the plant name, collection date, place of origin, and packaging date.

### 2.3. Essential Oil Extraction

The essential oil extraction process was carried out using the steam distillation method [32]. Briefly, 100 g of plant material was weighed into a round-bottom flask and 500 mL of water were added to the distillation apparatus (Clevenger distillation equipment) coupled to an electric heating mantle with temperature control up to 100 °C. Although each plant had its own particularities in the duration of the distillation process, the first 45 min after the start of boiling was sufficient to obtain most of the essential oil; the process was standardized for all plants used in 50 min, and at the end, the essential oil was collected in an amber glass bottle. The yield was measured, for this purpose, the essential oil-hydro residue was placed in 15 mL Eppendorf tubes, cooled to 4 °C and centrifuged for 10 min at 4000 rpm, and subsequently, the volume was collected and measured with a micropipette and placed in 1.5 mL Eppendorf tubes, and the oil was stored under refrigeration at 4 °C until later use. The distillation was repeated up to 20 times in plants with low productivity such as *C. citratus* and *C. verum*, which had the lowest productivity, and five times in the case of *S. molle*, which had the highest production.

### 2.4. Composition of Essential Oils

Gas chromatography–mass spectrometry (GC-MS) analyses were performed at the National Laboratory of Macromolecular Structure of the Center for Chemical Research of the Autonomous University of the State of Morelos (LANEM-CIQ-UAEM) using a 7890B gas chromatograph coupled to an Agilent Technologies 7000D triple quadrupole mass spectrometer, operated in EI mode at 70 eV (Agilent, Santa Clara, CA, USA). The oil samples were diluted in hexane (1:5, *v*/*v*), and 1 µL of each sample was injected. A 30 m long × 0.250 µm × 0.25 µm film thickness HP-5MS capillary column (5% diphenyl methylsiloxane) was used for separation. Helium (99.9%) was employed as the carrier gas at a constant flow rate of 1.3 mL/min. The injector and transfer line temperatures were set at 300 °C and 310 °C, respectively. The oven temperature program was as follows: initial temperature of 50 °C (held for 1 min), increased to 300 °C and held for 1 min, followed by a ramp of 10 °C/min to 310 °C, which was maintained for 20 min. The total run time was 49.78 min. Mass spectra were recorded as a function of retention time (RT, min) and relative abundance (%). The constituents were identified based on the retention index (RI) on the column, determined relative to homologous series of n-alkanes (C7–C30). The relative quantification of the compounds was based on the area under the curve of each peak by comparison of the obtained spectra with reference spectra from standard mass spectral libraries [33].

### 2.5. Larvae of R. microplus and A. mixtum

Fifteen-day-old larvae of *R. microplus* of the M-C strain, which exhibits low resistance to pyrethroids, were used. This strain was collected in 2014 in the municipality of Tapalpa, Jalisco, Mexico, and maintained at the CENID SAI, INIFAP facilities. For *A. mixtum*, fifteen-day-old larvae of hatching obtained ovipositions from engorgeous females collected from the natural infestation of a bovine in the URUSSE, UACh, were used, which were identified through dichotomous keys [34].

### 2.6. Larval Immersion Test (LIT)

Larvae of the two tick species were challenged to five EOs using the larval immersion test (LIT) [35]. A negative control (distilled water + 20% ethanol + 1% Tween 20) and three positive controls were also included. These positive controls consisted of the three main chemicals commonly used for tick control at the manufacturer’s recommended dosage. The chemical controls used were coumaphos (CO) at a dose of 0.2 mg/mL, flumethrin (FL) at a dose of 0.03 mg/mL, and amitraz (AM) at a dose of 0.2 mg/mL.

The oils were initially prepared in a solution consisting of 2.94 mL of distilled water + 0.04 mL of EO, + 0.22 mL of 1% Tween 20, + 0.80 mL of 20% ethanol, to achieve a final concentration of 1% of the oil (10 mg/mL). Six dilutions of the EOs were prepared from this solution to achieve concentrations of 10, 5, 2.5, 1.87, 1.25, and 0.6 mg/mL.

To evaluate the mortality of EOs, 300–450 tick larvae were initially collected using a brush and dissecting needle and placed in a 1.5 mL Eppendorf tube containing the previously diluted EO. The tube was capped, and the solution was homogenized with the larvae by shaking and allowed to stand for 10 min. The diluted EO was then removed, and approximately 100 larvae were extracted and placed in a 6 × 8 cm filter paper envelope, which was sealed with a snap fastener. This procedure was repeated for each EO concentration and its three replicates. Finally, each envelope was allowed to stand for 15–20 min to remove excess moisture and then incubated at a controlled temperature (27 °C) and 80% relative humidity, for 48 h. After that period, larval mortality was assessed by counting live and dead larvae [35]. The controls had no mortality, so the Abbott correction was not made.

### 2.7. Statistical Analysis

Using the live and dead larvae counts derived from the in vitro bioassays of each EO, the lethal concentration at 50% (LC_50_), the lethal concentration at 95% (LC_95_) and the lethal concentration at 99% (LC_99_) were calculated using the following logistic regression model (Probit) with the SAS 9.4 program [36].

$$\mathrm{Pr}\;(\mathrm{Response})=C+(1-C)\;F(x\prime \beta )=C+(1-C)\;\Phi \;(\beta_{0}+\beta_{1}\;\times \;\log10\;(\mathrm{Doses}))$$

where β is a vector of estimated parameters. F is a cumulative distribution function (Normal). X is a vector of explanatory variables. Pr is the probability of a response. C is the natural response rate (proportion of individuals that respond to zero dose). Φ: Normal cumulative distribution function.

## 3. Results

### 3.1. Production and Composition of Essential Oils

All plants used in this study produced EOs in varying amounts. The highest EO production was in *S. molle* seeds at 6.75%, while plants such as *C. citratus* have the lowest production at approximately 0.2% (Table 2).

The main components of *P. amboinicus* were Eucalyptol (27.2%) and Carvacrol (12.3%), for *C. verum* they were Eugenol (90.8%) and Caryophyllene (9.2%), in the case of *S. molle* the main components were D-Limonene (26.3%) and α-Phellandrene (23.3%), for *C. citratus* they were Citral (29.4%) and Geranial (25.9%), and in *P. dioica* the most abundant components were Methyleugenol (63%) and Eucalyptol (11.9%), as shown in Table 3. The proportions of each of the secondary metabolites of the five main plants used in tick larvae control are listed in Table 3.

### 3.2. Effect of Essential Oils on Tick Larvae

With essential oils, the highest mortality rates always occurred at the highest concentration used, which corresponded to a 1% dilution (10 mg/mL). Only flumethrin did not reach mortality values higher than 90% with the recommended commercial dose (Table 4).

### 3.3. Effect of Essential Oils on Rhipicephalus microplus

**Figure 1:**
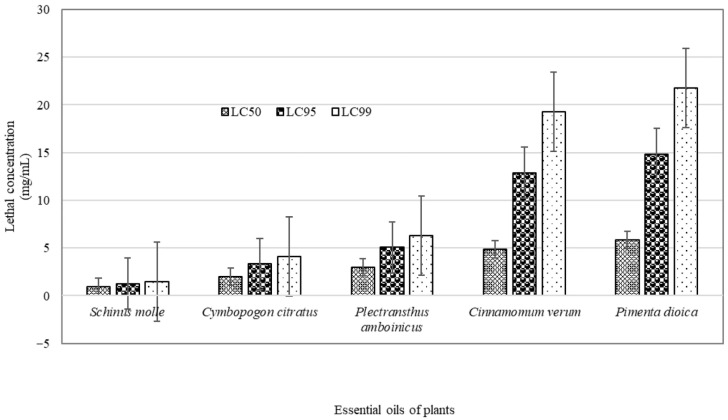
Lethal concentrations at 50% (LC_50_), 95% (LC_95_), and 99% (LC_99_) of the five essential oils used for the control of *Rhipicephalus microplus* larvae.

The most effective EO, with the lowest LC_50_ value in *R. microplus* larvae, was observed with the *S. molle* (0.92 mg/mL), while the highest LC_50_ (less effective) was recorded with the use of *P. dioica* (5.85 mg/mL), with a very wide difference between the two EOs. The EOs of *S. molle* and *C. citratus* were the most effective in causing mortality in *R. microplus* larvae, as they showed the lowest concentrations at which the highest mortality was obtained, while the EOs of *C. verum* and *P. dioica* required concentrations greater than 5 mg/mL to achieve mortality greater than 50% in *R. microplus* larvae (Figure 1).

Table 5 shows the mortality equations and lethal concentrations obtained from the Probit analysis for each EO. The most effective EO in causing mortality of *R. microplus* larvae was *S. molle*, with the lowest LC_50_ (0.92 mg/mL), and also with the lowest LC_99_ value of 1.43 mg/mL. The EOs used in this study had small LC_50_ values, ranging from 1.4 mg/mL to 5.85 mg/mL. *C. verum* and *P. dioica* had the highest concentrations.

The chemical controls behaved as expected, since the strain was moderately resistant to flumethrin (70% mortality), amitraz (100% mortality), and coumaphos (100% mortality).

### 3.4. Effect of Essential Oils on Amblyomma mixtum

**Figure 2:**
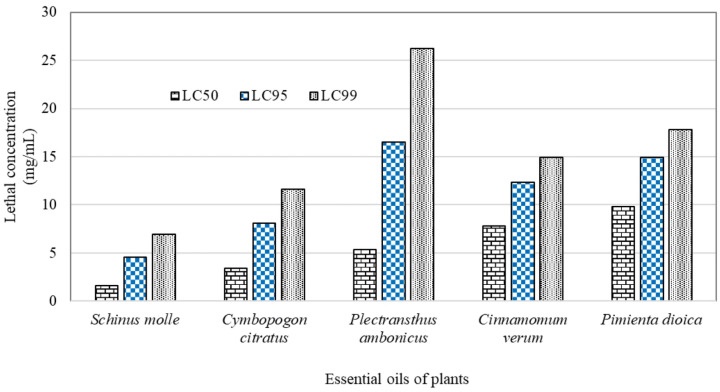
Lethal concentrations at 50%, 95% and 99% of five essential oils derived of aromatic plants used against *A. mixtum* larvae.

In *A. mixtum*, mortality induced by EOs exhibited a similar behavior to that observed in *R. microplus*. The lowest LC_50_ was observed with *S. molle* EO (1.6 mg/mL), while the highest concentration used was observed with *P. dioica* (9.81 mg/mL). *S. molle*, *C. citratus*, and *P. amboinicus* EOs were the most effective, as they showed the lowest concentrations required to achieve the highest mortality, while *C. verum* and *P. dioica* EOs required concentrations greater than 9 mg/mL to achieve mortality exceeding 50% in *A. mixtum* larvae (Figure 2).

Table 6 shows the mortality equations resulting from the PROBIT analysis for each oil. The EO with the highest effectiveness against *A. mixtum* larvae, was *S. molle*, with an LC_50_ of 1.60 mg/mL, and also with the lowest LC_99_ value of 6.97 mg/mL. The EOs used against *A. mixtum* in this study had small LC_50_ values, ranging from 1.60 mg/mL to 9.81 mg/mL. Meanwhile, *P. dioica* and *P. amboinicus* had the highest concentrations.

The chemical controls performed as expected, as the ticks showed no resistance to the chemical controls flumethrin (90% mortality), amitraz (92% mortality), and coumaphos (98% mortality) because the cattle from which it was obtained are not frequently bathed with these products.

## 4. Discussion

The use of plant-derived secondary metabolites has been one of the control alternatives for internal parasites, including ruminant nematodes [37,38,39], equine cyathostomins [40], and for the control of external parasites such as ticks [29,41]. Many plant components have been tested in vitro with encouraging results, and in vivo evaluations of many products, especially those that have been extensively studied, are currently underway [42]. However, given the large number of products with parasiticidal activity, in vitro evaluation is initially required to determine the median lethal dose and thus identify a candidate product for use in vivo trials.

In this study, all EOs from the evaluated plants showed dose dependence, with mortality increasing as the EO dose increased. Of the five EOs studied, *S. molle* performed best, achieving the lowest LC_50_ values of 0.9 and 1.6 mg/mL in *R. microplus* and *A. mixtum*, respectively. In addition to its use as an EO, it has been applied as an extract dissolved in water in both in vivo and in vitro tests against the tick *R. microplus*, yielding encouraging results at high concentrations. In vitro tests indicated 100% mortality in larvae ticks at a concentration of 10% [43]. The same EO also showed 100% mortality in non-ingurgid *R. microplus* larvae at a concentration of 5%. Similar results were observed with the EO of *Bursera graveolens* [27]. Furthermore, using the crude extract of *S. molle* on ticks of the species *Rhipicephalus pulchellus* and *Boophilus decoloratus*, a 57% mortality rate was found at a dose of 1%, 70% at 2%, and 100% at a concentration of 4% [44]. All these studies have shown high efficacy, but with high concentrations, unlike the results of the present study, in which lower concentrations were required to achieve an LC_99_ (1.4 mg/mL or 0.14%) in *R. microplus* larvae, demonstrating an acaricidal potential.

For the *C. citratus* EO, the LC_50_ for *R. microplus* was 1.97 mg/mL (LC_95_ = 3.32 and LC_99_ = 4.13 mg/mL), while for *A. mixtum* it was 3.44 mg/mL (LC_95_ = 8.12 and LC_99_ = 11.58 mg/mL). These values were within the range observed in a study in Brazil against *R. microplus* (LC_50_ = 5.65 and LC_90_ = 7.12 mg/mL) [29] and similar to another study using the same species with the larval packet test [45]. Mortality reports in adult *R. microplus* have also shown that microemulsions at concentrations greater than 5% result in approximately 99% mortality [46]. This oil has also been used to control the longhorned tick (*Haemaphysalis longicornis*), which has been observed in Asia, Australia, and New Zealand, achieving up to 94% larval mortality with a concentration of 40 mg/mL [47]. Another study considering the synergistic effect of *Eucalyptus globulus* and *Cymbopogon flexuosus* also showed high mortality at concentrations of 4% [48]. In Malaysia, the LC_50_ and LC_90_ for *C. citratus* EO were calculated to be 1.21% and 6.28%, respectively, using the LIT and readings taken at 24 h, while at 48 h, the results were 1.05% and 6.12% [49]. Because high tick larval mortality is achieved with only 4.13–11.58 mg/mL, this oil is a candidate for use in the control of *R. microplus*, and for this reason, several studies have been carried out showing its effect on ticks and developing a self-emulsifying drug delivery system to enhance stability and efficacy [46].

Some studies with *R. microplus* show that *C. verum* is effective in controlling this tick with the use of small doses, such as the case in which a dose of 0.86 mg/mL of *C. verum* EO was reported to achieve 50% larval mortality and with values of 1.27 mg/mL 99% larval mortality was achieved [45], while another study with a concentration of 2.5 mg/mL (0.25%) of *Cinnamomum zeylanicum* achieved 100% mortality in *R. microplus* larvae [50]. In the present study, a higher dose than that reported in the literature was required, and the observed values were 4.86 mg/mL to achieve 50% mortality of *R. microplus* larvae and 19.27 mg/mL (1.9%) to achieve LC_99_. However, another study in *H. longicornis* found a higher LC_50_, with values of 16.07 mg/mL (9.66–25.92 mg/mL) in unfed larvae and 77.28 mg/mL (70.00–85.24 mg/mL) in fed adults, and in adults and larvae of *R. microplus*, the values obtained were 5–6% to achieve LC_50_ [51]. The fumigant toxicity test showed significant acaricidal activity against both unfed and engorged nymphal and adult *H. longicornis*. Enzyme assays revealed that the EOs of both *C. verum* significantly inhibited glutathione S-transferase activity (*p* < 0.05) [52]. Several studies have indicated that the main component of *C. verum* is trans-cinnamaldehyde (49.42%) [51,52] as well as other components such as benzyl benzoate (20.9%), β-caryophyllene (13.5%), linalool (9.4%), and eugenol (6.70%) [52], unlike what was observed in the present study in which the main component was eugenol (90.8%) and caryophyllene (9.2%).

Another EO that also showed a highly effective dose that reported in other studies was peppercorn oil (*P. doica*), which, at a concentration of 1.26%, achieved 100% mortality for *R. microplus* larvae [53]. The pepper essential oil evaluated in this experiment required a high dose of 21.77 mg/mL (2.17%) to achieve an LC_99_. The *P. dioica* EO used in this experiment required more than 5.85 mg/mL to achieve mortality exceeding 50% in the *R. microplus* population and more than 9.81 mg/mL in *A. mixtum*. This EO has been extensively studied, even in agriculture, for the control of mites such as *Tetranychus tumidus*, in evaluations with concentrations of 1% [54].

In the case of *P. amboinicus* in Brazil, the use of this EO has been reported for the control of engorged female *R. microplus* at a concentration of 1%, with an efficacy of 98% in inhibiting the reproductive rate of this species [30]. A 10% mortality rate has been reported in *R. microplus* larvae evaluated using the larval pack test with a 5% of EO concentration [55]. There are also reports of the use of this essential oil against insects such as the weevil (*Tribolium castaneum*) [56]. No studies were found on the use of this essential oil against *A. mixtum*; therefore, there is an area of opportunity for research with larvae, nymphs or adult ticks and the inhibition of reproductive parameters.

Of the secondary metabolites, essential oils have been a widely used option for tick control [57]. The high effectiveness of essential oils has been attributed to the fact that their compounds inhibit the tyramine receptor, inhibit the acetylcholinesterase enzyme, and modulate the GABA receptor [58]. Therefore, it has been indicated that the effect of essential oils is rapid, and mortality can be assessed within 24 h of application [54]. Among the results reported by some authors using EO, cellular damage in the ovaries and salivary glands of *R. microplus* has been analyzed and attributed to thymol [59]. Some compounds in EO are attributed to affecting octopamine receptors (tyramine and β-adrenergic receptors) and their alteration causes a complete collapse of the nervous system in insects [53].

Compounds such as (E)-cinnamaldehyde have been used as an enhancer of commercial products like amitraz or chlorfenvinphos, increasing mortality in *R. microplus* larvae from 50% to 80% [60], a value that is also considered high.

The main component of *S. molle* was D-limonene, followed by α-Phellandrene and β-Pinene, which, in addition to causing high mortality in *R. microplus* larvae in this study, can affect reproductive values such as egg viability and morphological changes in various tissues, including female reproductive organs [61]. In the case of *C. citratus*, the main components were citral and geranial, as well as β-Myrcene. In the case of *P. amboinicus*, the main components were eucalyptol, carvacrol, and α-Phellandrene. The combined effect of thymol, carvacrol, and (E)-cinnamaldehyde on larvae of the ticks *Amblyomma sculptum* and *Dermacentor nitens* have also been reported; these compounds are found in the EO of cinnamon [62]. Thymol and carvacrol, when interacting with muscarinic acetylcholine receptors (mAChRs), act as agonists and activate the receptors, causing smooth muscle relaxation or modulation of glandular secretions [63].

For *C. verum*, the main component was Eugenol followed by Caryophyllene, while for *P. dioica*, Methyleugenol, eugenol and Eucalyptol were the main compounds found [64]. Eugenol can bind with acetylcholine receptors, inhibit their activity, and cause paralysis of *Hyalomma scupense*.

These findings identify *S. molle*, *C. citratus*, *P. amboinicus*, *C. verum*, and *P. dioica* as promising candidates for the development of plant-based acaricides, supporting their potential integration into the sustainable tick management strategies for management of acaricide-resistant *R. microplus* and *A. mixtum* populations.

Although the results obtained are encouraging, further studies are needed on adult ticks, which disperse a large number of offspring in the environment and in which there are few studies on the effects of essential oils [26].

Despite the large number of properties attributed to essential oils due to their mixture of components, further study is needed to understand their effect on the host and any potential side effects. Recent reviews address some important aspects to consider for their use in vivo animal testing [65].

## 5. Conclusions

The essential oils (EOs) of the plants evaluated (*S. molle*, *C. citratus*, *P. amboinicus*, *C. verum*, and *P. dioica*) in this study showed a dose-dependent effect, exhibiting higher mortality rates as the doses of the five oils used increased. Although this represents a potential alternative for the control of *R. microplus* and *A. mixtum*, these preliminary results need to be confirmed due to the great variability in plant composition caused by intrinsic and environmental effects.

The essential oil of *S. molle* was the most effective product, causing 50% mortality at a concentration of 0.92 mg/mL for *R. microplus* and 1.6 mg/mL for *A. mixtum*. The essential oil of *P. dioica* showed the lowest efficacy against *R. microplus* larvae, requiring doses greater than 10 mg/mL to achieve mortality exceeding 50%. In contrast, against *A. mixtum*, the lowest acaricidal effect was observed with the EO of *P*. *amboinicus*, requiring concentrations greater than 16.5 mg/mL to achieve over 95% mortality.

The difference between the two species of the genus *Ixodes* was minimal in terms of the essential oil concentrations required for 99% mortality across the five plants on which the same essential oils were evaluated, regardless of whether one was an isolated and characterized strain and the other a tick collected in the field.

At least one major component was found in each of the essential oils, which possibly plays a role in the observed acaricidal activity.

## Figures and Tables

**Table 1:** Origin, anatomical structure and scientific name of the plants used to obtain essential oils.

Common Name	Scientific Name	Catalog Collection *	Anatomic Structure Used	Collection Site
Peruvian pepper tree	*Schinus molle* L.	673,428	Fruit	Temascalapa, State of Mexico
Lemongrass	*Cymbopogon citratus* (DC.) Stapf	1,201,181	Leaves	Salto de Agua, Chiapas
Mexican mint	*Plectranthus amboinicus* (Lour.) Spreng.	1,038,621	Leaves	Teapa, Tabasco
Cinnamon	*Cinnamomum verum* J. Presl	1,396,171	Leaves	Salto de Agua, Chiapas
Peppercorns	*Pimenta dioica* (L.) Merr.	762,667	Fruit	Teapa, Tabasco

* Catalog number derived from comparison with the repository of the National Herbarium of Mexico: https://ib.unam.mx/ib/colecciones-biologicas/herbario-nacional (accessed on 10 March 2026).

**Table 2:** Percentage of essential oil yield from the five selected aromatic plants and content of major compounds.

Common Name	Scientific Name	Oil Yield (%)	Main Component	Molecular Weight
Peruvian pepper tree	*Schinus molle*	6.75	D-Limonene	136
Lemongrass	*Cymbopogon citratus*	0.20	Citral	152
Mexican mint	*Plectranthus amboinicus*	1.00	Eucalyptol	154
Cinnamon	*Cinnamomum verum*	0.60	Eugenol	204
Peppercorns	*Pimenta dioica*	1.50	Methyleugenol	178

**Table 3:** Secondary metabolites, retention time and percentage content in the peak area found in the essential oils of five plants used in tick control.

Retention Time (Rt, min)	Compound	*Plectranthus amboinicus*	*Cinnamomum verum*	*Schinus molle*	*Cymbopogon citratus*	*Pimenta* *dioica*
		Abundance	Abundance	Abundance	Abundance	Abundance
		(%)	(%)	(%)	(%)	(%)
6.2	α-Pinene	4.9	-	11.1	-	-
6.45	Camphene	3.7	-	-	-	-
7.14	β-Myrcene	6.1	-	-	15.7	3.9
7.14	β-Pinene	-	-	18.9	-	-
7.36–7.39	α-Phellandrene	8.5	-	23.3	8.4	5.2
7.71	O-cymene	-	-	-	3.2	-
7.78	D-Limonene	-	-	26.3	-	-
7.82	Eucalyptol	27.2	-	-	-	11.9
9.62	(+)-2-Bornanone	3.2	-	-	-	-
9.94	Endo-Borneol	3.5	-	-	-	-
10.11	Terpinen-4-ol	6.3	-	-	-	-
10.31	α-Terpineol	4	-	-	-	-
11.05	Geranial	-	-	-	25.9	-
11.21	Geraniol	-	-	-	5	-
11.48	Citral	-	-	-	29.4	-
11.72	Thymol	5.6	-	-	-	-
11.87	Carvacrol	12.3	-	-	-	-
12.77	Eugenol	-	90.8	-	-	11
13.34	Methyleugenol	-	-	-	-	63
13.55	Caryophyllene	3.7	9.2	5.7	-	5
13.99	Humulene	2.5	-	-	-	-
14.33	Germacrene D	-	-	6.6	-	-
14.8	β-Cadinene	-	-	8.1	-	-
15.61	Caryophyllene oxide	2.6	-	-	-	-
16.4	β-Eudesmol	6	-	-	-	-

**Table 4:** Percentage of maximum mortality achieved, and doses used of the products, in tick larvae of two species.

Product	*Rhipicephalus microplus*		*Amblyomma mixtum*	
	Concentration (mg/mL)	Mortality %	Concentration (mg/mL)	Mortality %
*Cinnamomum verum*	10	91	10	90
*Cymbopogon citratus*	5	100	10	72
*Pimenta dioica*	10	93	10	56
*Plectranthus amboinicus*	7	100	10	92
*Schinus molle*	1.8	100	5	100
Amitraz (Positive control)	0.2	100	0.2	92
Coumaphos (Positive control)	0.2	100	0.2	98
Flumethrin (Positive control)	0.03	70	0.03	90

**Table 5:** Equations resulting from the Probit analysis, equation parameters, LC_50_, LC_95_ and LC_99_ and confidence intervals (CI, 95%) for each essential oil.

Essential Oil	Equation	β_0_	β_1_	LC_50_	CI (95%)	LC_95_	CI (95%)	LC_99_	CI (95%)
*Schinus molle*	12.55 + 12.12 × (Log(doses))	**	**	0.92	0.88–0.95	1.26	1.21–1.32	1.43	1.36–1.51
*Cymbopogon citratus*	5.11 + 7.24 × (Log(doses))	**	**	1.97	1.90–2.04	3.32	3.13–3.59	4.13	3.80–4.58
*Plectranthus amboinicus*	3.76 + 7.2 × (Log(doses))	**	**	2.99	2.89–3.12	5.07	4.74–5.49	6.31	5.80–6.97
*Cinnamomum verum*	1.22 + 3.88 × (Log(doses))	**	**	4.86	4.61–5.15	12.88	11.6–14.5	19.28	16.9–22.5
*Pimenta dioica*	0.94 + 4.97 × (Log(doses))	**	**	5.85	5.56–6.18	14.82	13.4–16.6	21.78	19.2–25.2

** β_0_: y-intercept passes through zero (*p* < 0.05) and ** β_1_: Slope of the line is different from zero (*p* < 0.05).

**Table 6:** Equations resulting from the Probit analysis, equation parameters and LC_50_, LC_95_, LC_99_ and confidence intervals (CI, 95%) for each essential oil in *Amblyomma mixtum*.

Essential Oil	Equation	β_0_	β_1_	LC_50_	CI (95%)	LC_95_	CI (95%)	LC_99_	CI (95%)
*Schinus molle*	0.90 + 8.24 × (Log(doses))	**	**	1.60	1.52–1.68	4.53	4.13–5.04	6.97	6.15–8.06
*Cymbopogon citratus*	2.05 + 4.41 × (Log (doses))	**	**	3.44	3.28–3.60	8.12	7.47–8.90	11.58	10.43–13.05
*Plectranthus amboinicus*	0.91 + 3.38 × (Log(doses))	**	**	5.37	5.11–5.64	16.48	15.65–17.30	26.21	24.90–27.52
*Cinnamomum verum*	0.90 + 8.24 × (Log(doses))	**	**	7.78	7.47–8.09	12.31	11.65–13.14	14.89	13.89–16.20
*Pimenta dioica*	0.08 + 9.01 × (Log(doses))	**	**	9.81	9.45–10.16	14.93	13.96–16.35	17.78	16.25–20.10

** β_0_: y-intercept passes through zero (*p* < 0.05) and ** β_1_: Slope of the line is different from zero (*p* < 0.05).

## Data Availability

The original contributions presented in this study are included in the article. Further inquiries can be directed to the corresponding author.

## Abbreviations

AM	Amitraz
CO	Coumaphos
EOs	Essential oils
FL	Flumetrhin
GABA	γ-aminobutyric acid
LC	Lethal concentration
LIT	Larval immersion test
